# Supplementary material for: Distinct contributions by frontal and parietal cortices support working memory
Source: Sci Rep. 2017 Jul 21;7:6188. doi: 10.1038/s41598-017-06293-x (PMC5522403; doi:10.1038/s41598-017-06293-x)
Supplement: Supplementary file 1 — Supplementary Information [file 41598_2017_6293_MOESM1_ESM.pdf]

Supplemental Materials for

Distinct contributions by frontal and parietal cortices support working memory

Wayne E. Mackey[1] and Clayton E. Curtis[1,2,*]
[1] Department of Psychology, New York University
[2] Center for Neural Science, New York University
New York University, 6 Washington Place, New York, NY 10003, USA
[*]Correspondence: clayton.curtis@nyu.edu

Supplemental Figures

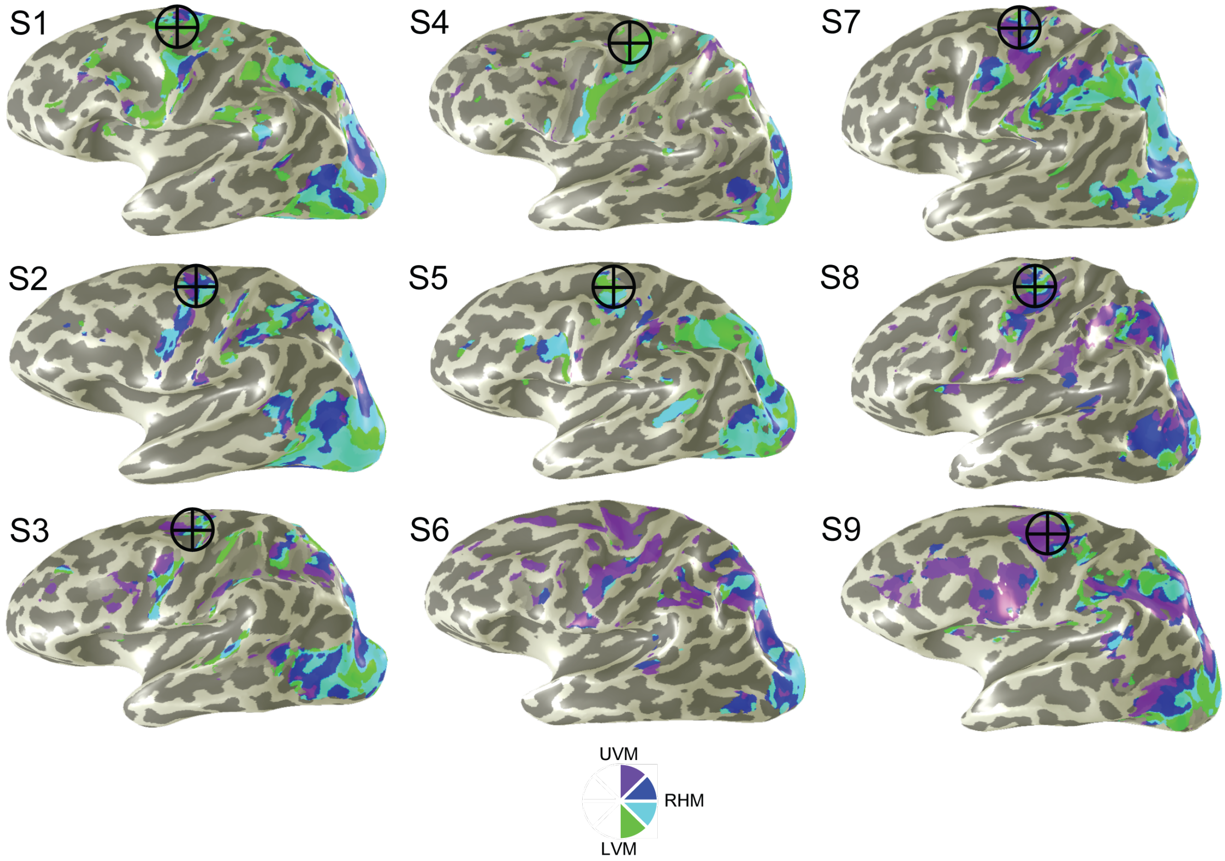

Figure S1 Retinotopic mapping and PCS stimulation sites. Phase angle maps for each individual subject are projected onto an inflated cortical surface of the left hemisphere. The color of each voxel indicates the preferred phase angle of that particular voxel. sPCS stimulation sites are indicated by black crosshairs. While phase maps are shown for each individual subject, S6 did not perform a session with stimulation to PCS, so no crosshairs are shown.

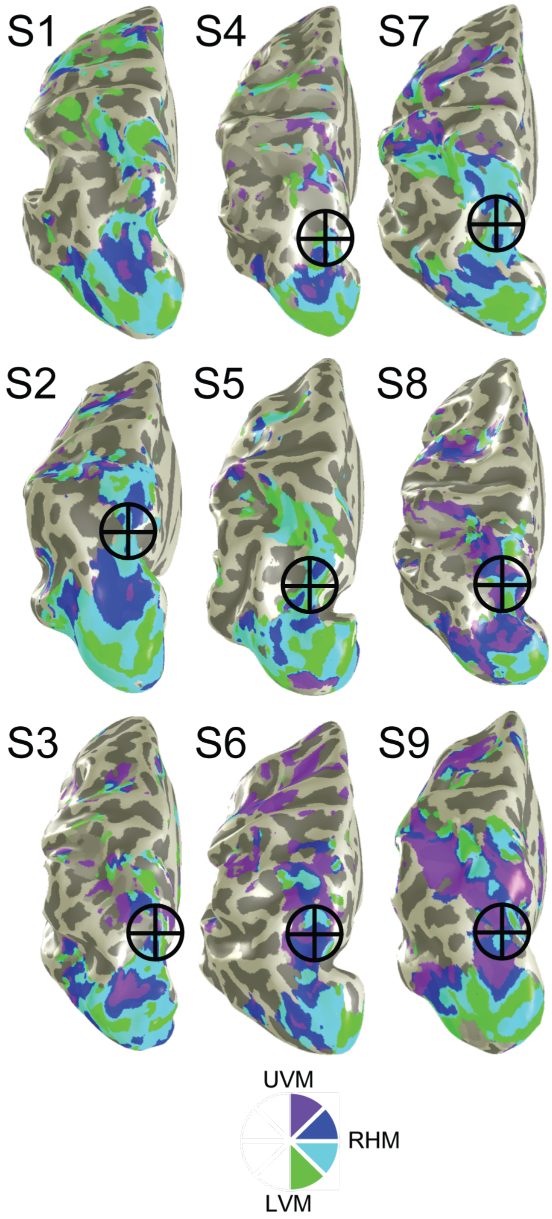

Figure S2 Retinotopic mapping and IPS stimulation sites. Phase angle maps for each individual subject are projected onto an inflated cortical surface of the left hemisphere. The color of each voxel indicates the preferred phase angle of that particular voxel. IPS2 stimulation sites are indicated by black crosshairs. While phase maps are shown for each individual subject, S1 did not perform a session with stimulation to IPS, so no crosshairs are shown.

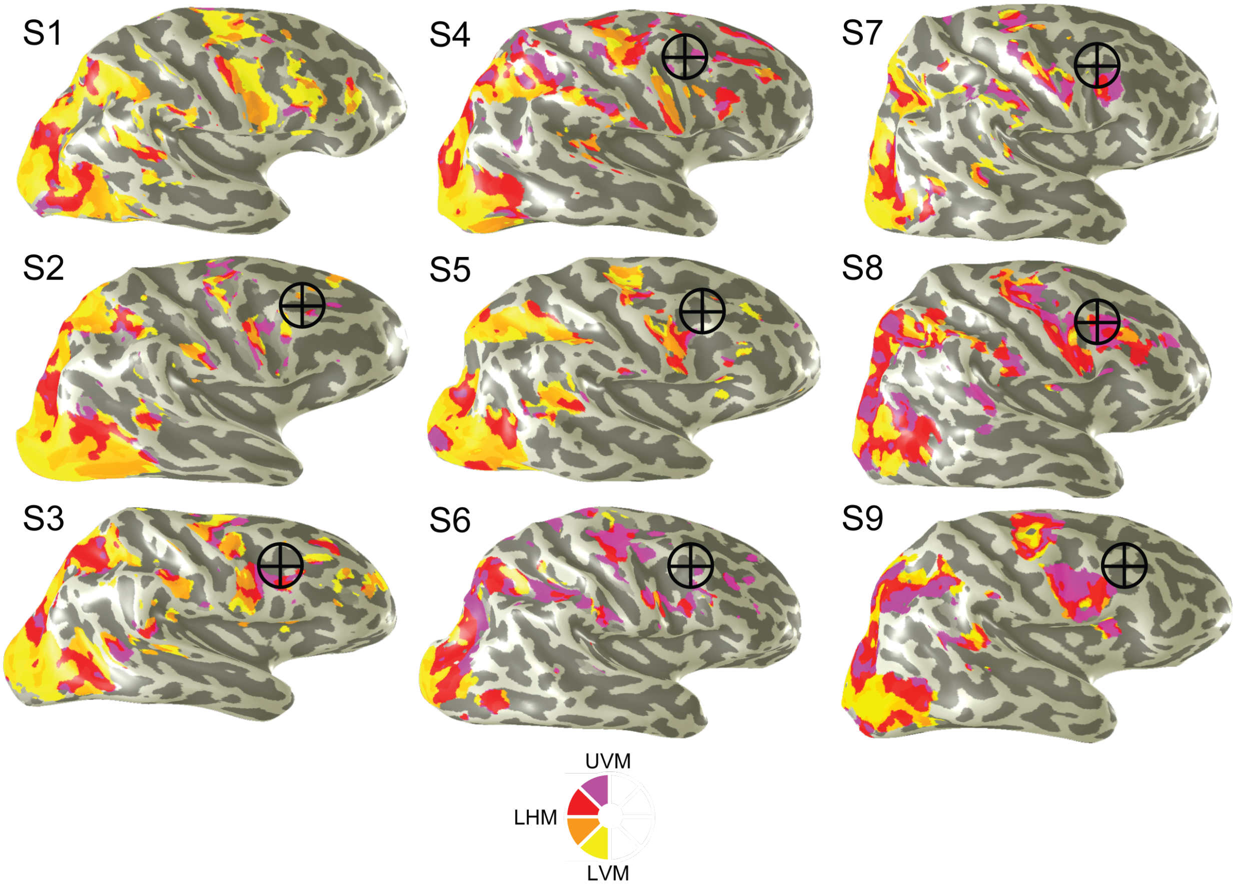

Figure S3 Retinotopic mapping and PFC stimulation sites. Phase angle maps for each individual subject are projected onto an inflated cortical surface. The color of each voxel indicates the preferred phase angle of that particular voxel. PFC stimulation sites are indicated by black crosshairs. Note that for most subjects, no coherent map of phase angle is observed in the dorsolateral PFC. Therefore, individual dorsolateral PFC stimulation sites were chosen based on previous anatomical studies. While phase maps are shown for each individual subject, S1 did not perform a session with stimulation to PFC, so no crosshairs are shown.

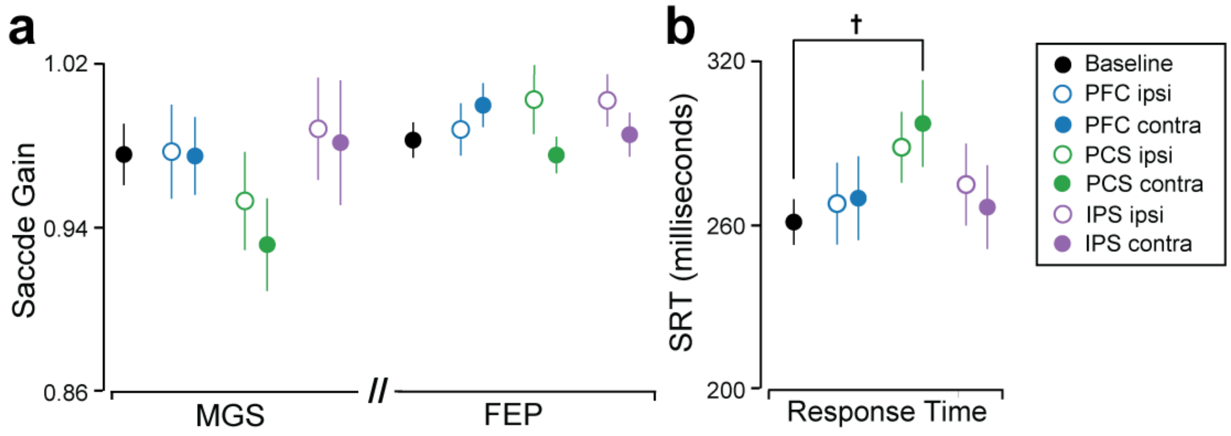

Figure S4 Gain and SRT group results. **†** p <= 0.08. Error bars represent standard error of the mean.

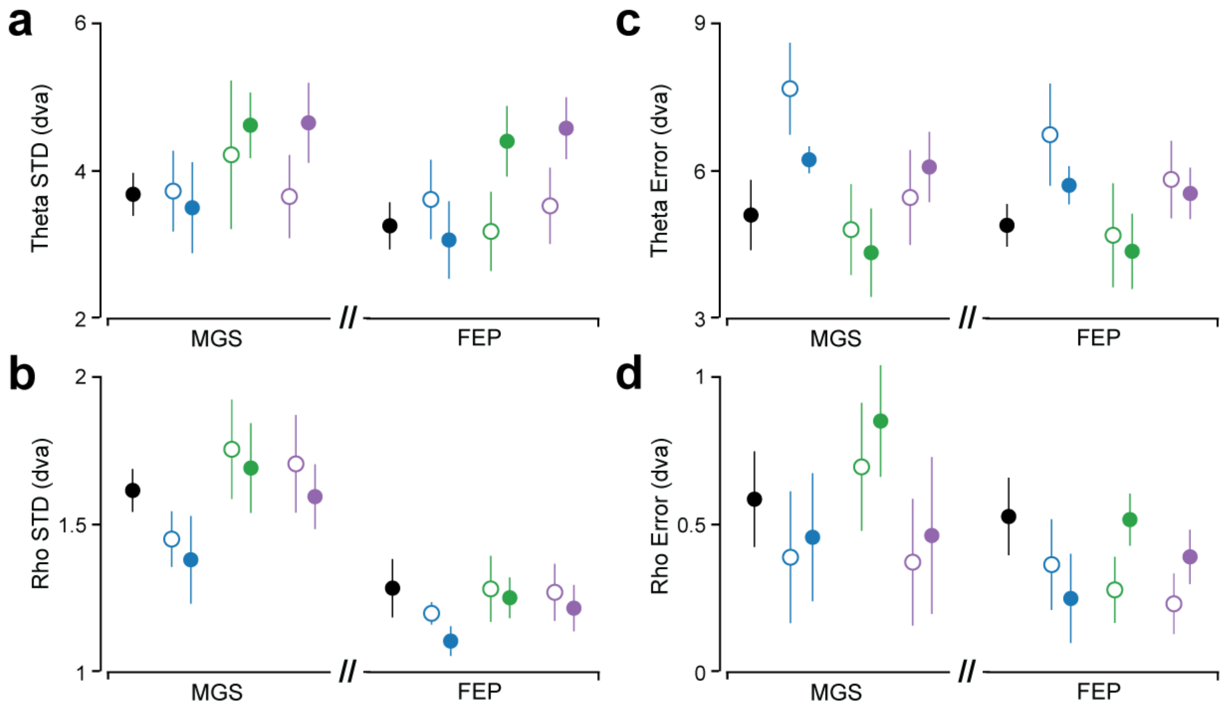

Figure S5 Error and STD in polar coordinates theta and rho. Error bars represent standard error of the mean. No significant differences were found, suggesting no affect of systematic bias as a consequence of TMS. Same group legend as Figure S4.

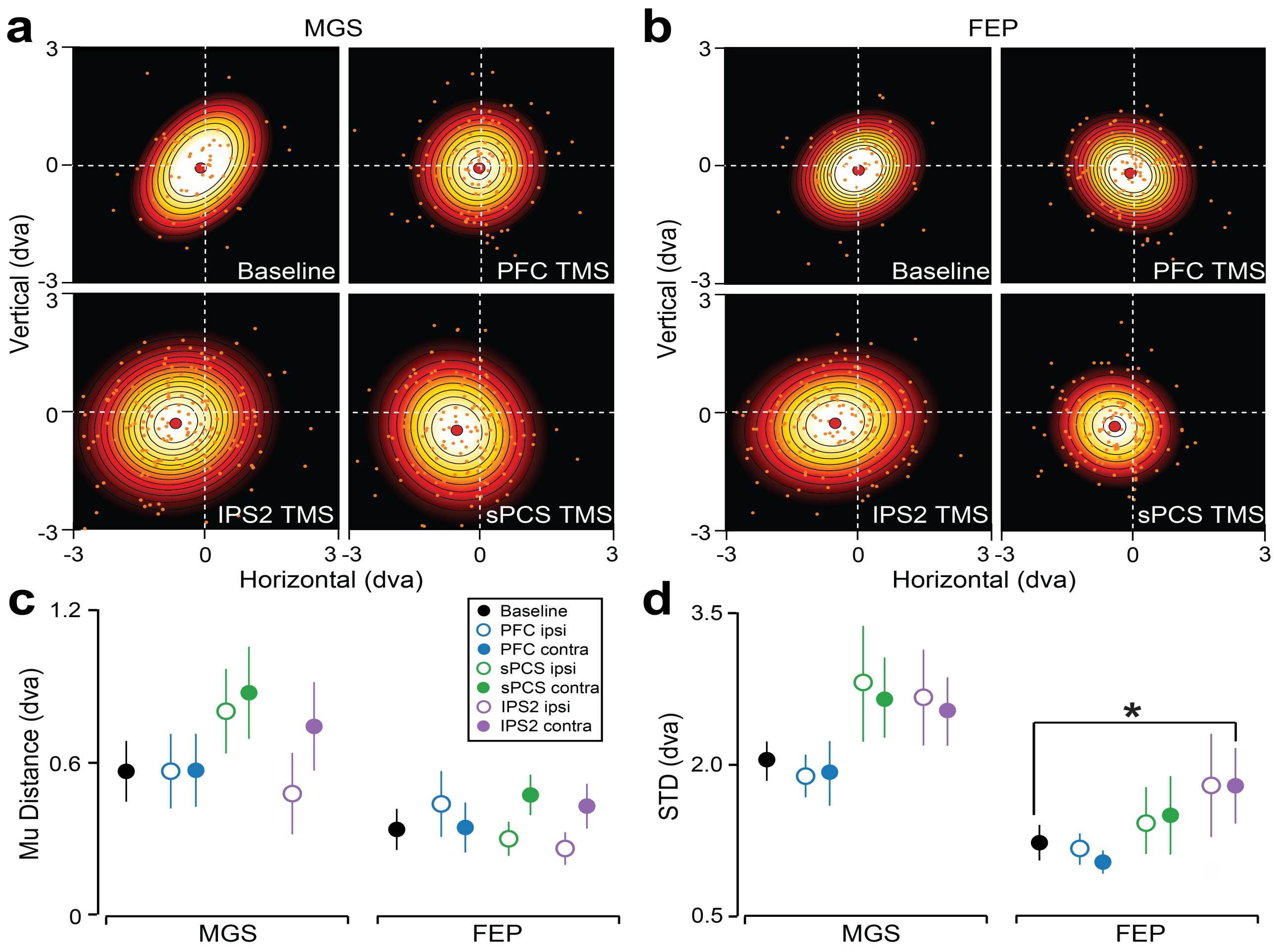

Figure S6 Patterns of systematic error after frontal and parietal disruption. Data from a single subject is shown for both (***a***) memory-guided saccades (MGS) and (***b***) final eye position (FEP). Individual trial target locations were transformed to a single location, then saccade endpoints and gaze positions (orange dots) were fit with a single component Gaussian mixture distribution model displayed in each plot. (**c**) Changes in the mean and (**d**) standard deviation of the best fit Gaussian for each condition for both MGS and FEP. Error bars represent the standard error of the mean. * $p <= 0.05$.

Supplementary Discussion

No systematic spatial distortions after TMS

We tested if differences existed in the spatial distribution of error after frontal and parietal stimulation. Since we found no obvious bias in error in saccade gain, a typical measure of hypo or hypermetria **(Fig. S4a)**, we investigated systematic spatial effects in two other ways. First, we looked at components of saccade error, theta and rho, individually. We found so significant differences in the mean or variability across stimulation sites for either component **(Fig. S5)**. Second, we fit a model to the 2D error distribution to quantify changes in the mean and variability of this distribution as a function of TMS. To quantify any differences, we collapsed all trials within one half of the visual field to a single location. We then fit a Gaussian to the saccade endpoints relative to the collapsed target location for both primary and final saccades. This allowed us to investigate the nature of the spatial error by looking at changes in both the mean and the standard deviation of the 2D Gaussian fit to the saccade endpoints for each TMS condition. Again, we found no clear evidence for a systematic spatial bias or increase in variability (**Fig. S6**). Thus, the WM errors caused by TMS are spatially random and cannot be reduced to a single component measure of spatial distortion.
